# Vascular Biomarkers: Physics Parameters and Circulating Molecules Can Be Two Faces of the Same Coin

**DOI:** 10.3390/diagnostics11020217

**Published:** 2021-02-02

**Authors:** Paolo Zamboni

**Affiliations:** Department of Surgery, Vascular Disease Centre University Hospital of Ferrara, 44124 Cona, Italy; paolozamboni@icloud.com; Tel.: +39-05-3223-7694

The arterial, venous and lymphatic conduits of human circulation are a fascinating field of research. The Special Issue of *Diagnostics* dedicated to vascular biomarkers challenges the classic medical vision that considers as biomarkers almost exclusively the molecules circulating in the blood. Recent investigations, aimed at understanding the contribution of flow to atherosclerosis and chronic venous disorders, have already demonstrated the direct relationship between haemodynamic forces and endothelial cytokines expression: While a laminar flow is associated with low inflamed vessel walls, an oscillatory flow is linked to a pro-inflammatory endothelial lining (Figure 1) [1].

This Special Issue contains innovative articles describing physics markers linked to blood flow haemodynamics and to oxygenation and fluids distribution in the tissues. It appears clear that physical parameters of blood flow, blood oxygenation, imaging patterns, and electric signaling as well as the impedance linked to the distribution of water in the body contain innovative and useful levels of information in clinical practice. 

The common denominator of the articles in this Special Issue is the authors’ proposal to use for diagnostic purposes energies that we could define “green”, completely avoiding the use of ionizing radiation. This allows them to obtain very interesting results using techniques that are really not harmful.

It is the case of Near-Infrared Spectroscopy (NIRS) a versatile optical noninvasive technology more commonly used in peripheral arterial disease (PAD) and/or in diabetes [2,3]. NIRS allows assessing, respectively, oxy and deoxyhemoglobin directly in the tissues, giving an immediate and precise lecture of the oxygen exchanges at the level of microcirculation [4].

Bonilauri et al. addressed a systematic review on the use of the NIRS technology for investigating cortical haemodynamic activity in the most common chronic neurological conditions, such as Parkinson’s Disease, Alzheimer’s Disease, Mild Cognitive Impairment, and Multiple Sclerosis (MS) [5].

The latter, being the most common chronic neurological disease in young adults, attracted other authors who explored the vascular aspects of MS [6,7]. Unconventional brain magnetic resonance techniques make it possible to transform such diagnostic tools into a sort of noninvasive external laboratory. This is the case of two very high-level papers that have been proposed by the Buffalo Neuroimaging Analysis Center [8,9]. The first article explores longitudinally the presence of cerebral microbleeds in patients suffering from MS. The authors use very sophisticated MR techniques, such as susceptibility weighted imaging, capable of demonstrating the presence of such a novel microvascular aspect in MS patients. However, their data still do not permit to understand why these phenomena develop around the cerebral venules, or if heme iron derived from microbleeding could be the major contributors to perivenular iron stores seen in MS [10].

Very intriguingly, in the second MR article the authors investigated the relationship between the perfusion of the thalamus and a serum biomarker of inflammation and neurodegeneration in MS, the neurofilament light [9].

Both imaging and blood biomarkers are known to be independently associated with MS disability. The authors demonstrated a robust relationship between lower thalamic normalized cerebral blood flow and volume with greater serum neurofilament levels, strengthening the role of the vascular component in MS pathogenesis. Furthermore, this article suggests that imaging and circulating biomarkers can be two faces of the same coin. In addition, Pirastru and co-workers investigated by the means of MRI the cerebral perfusion. In healthy subjects they assessed brain perfusion in relation to the individual inflow via the internal carotid artery. They identified sort of cerebral hub perfusional areas related to functional networks, besides vascular territories such as that of the internal carotid artery. It could be hypothesized the perfusional hub areas could be altered in neurodegenerative diseases. The research is promising but still evidently in progress [11].

The effects of exercise on the vascular apparatus represent a second block of papers of this Special Issue. Menegatti and collaborators explored for the first time the effects of aquatic exercise on cellular and extracellular water distribution. It is known that aquatic exercise, by increasing the external component of the transmural pressure, is beneficial for tissue drainage, but now the bio-impedance assessment adds a novel tool that can be used in the field to monitor the results of the vascular fitness in the water [12].

Manfredini and co-workers, by using the term “training prescription”, permit readers to understand how in the future of patients, especially with co-existing vascular problems, exercise intensity and modality needs more science behind it in order to transform the training into a powerful drug. More precisely, mitochondrial biomarkers demonstrated the superiority of personalized gait training in respect to exaggerated exercise training prescribed in primary progressive MS patients [13].

Moreover, the Special Issue also includes other more traditional but excellent articles, exploring the significance of circulating molecules either in PAD or in myocardial infarction in order to improve our understanding and prognosis of cardiovascular disease [14,15].

The last block of articles published in Volume 1 of the Special Issue represents the increasing evidence of the endothelial cells’ attack on behalf of the novel Sars-Cov2 virus, maybe representing the major cause of mortality of the pandemic developed worldwide in 2020. Covid-19 is a respiratory disease with more than 80% asymptomatic patients, with a still-not-explained state of hypercoagulability that leads to a number of venous thromboembolic and other cardiovascular complications affecting both morbidity and mortality [16,17,18]. 

Preliminary and precious data from a postmortem study published in the Special Issue demonstrates the endothelial injury at the level of the lung as well as the hyperexpression of Factor VIII, both supporting the vascular involvement in Covid-19 disease [19].

The high quality and the number of the manuscripts submitted to the present Special Issue support the editorial decision to open a second Special Issue in 2021.

## Figures and Tables

**Figure 1:**
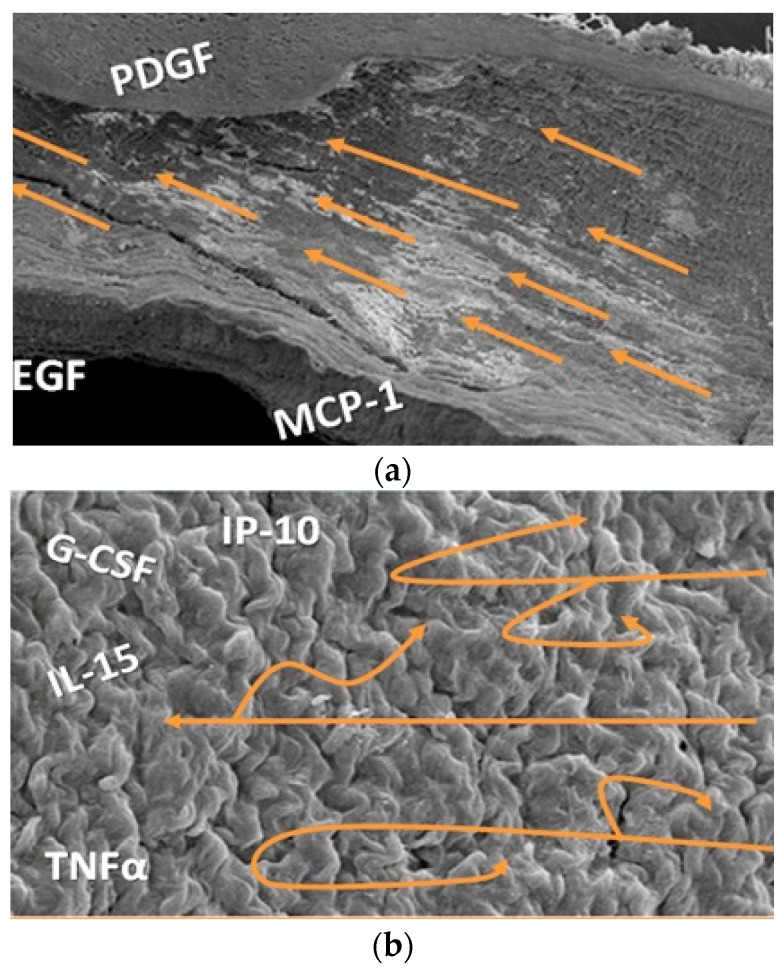
Scanning electronic microscopy of the lumen of human veins. On the top (**a**) regular disposition of the endothelial cells in accordance with direction of the laminar flow regimen. Bottom (**b**): irregular arrangement of the endothelial cells in response to a turbulent and oscillatory flow regimen. The labels refer to the different endothelial expression of cytokines respectively induced by laminar flow, top, and by turbulent and oscillatory flow regimen, Bottom.
